# Understanding the ideal resources to study the UKMLA

**DOI:** 10.1186/s12909-026-08814-7

**Published:** 2026-02-23

**Authors:** Parth Ankur Tagdiwala, Christina Anna Petmeza, Sanskritti Dubey, Inez Murray, Arisma Arora, Nikki Kerdegari

**Affiliations:** 1 University College London Medical School, Gower St, London, WC1E 6BT UK; 2 Queen Mary University of London, 327 Mile End Road, London, E1 4NS UK; 3 The University of Nottingham, Nottingham, NG7 2RD UK; 4 Queen’s University Belfast, University Road, Belfast, BT7 1NN Northern Ireland UK; 5 Kings College London, King’s College London Strand, London, WC2R 2LS UK

**Keywords:** E-learning, Learning resources, Question banks

## Abstract

**Background:**

The United Kingdom Medical Licensing Assessment (UKMLA) is a new national examination being introduced for all final-year medical students graduating from the academic year commencing in 2024. The examination will be a national initiative to standardize medical school exams. In this paper, we aim to understand which resources can be developed in the future to help clinical-year medical students studying for the UKMLA.

**Methods:**

We organised a novel 25-lecture series delivered by post-CCT doctors using an online platform. A form was created and distributed amongst the lecture audience to understand which resources medical students find useful for studying clinical medicine. The form was created using Google Forms©.

**Results:**

Our form was completed by 71 participants. The three most used resources were free online resources, paid question banks, and clinical placements. More than half of the participants reported that the single most useful resource was paid online question banks. The form was completed by participants from a wide range of UK medical schools, with most students being in their clinical years of study.

**Conclusion:**

Digital resources are widely used by medical students, and to further support clinical students in their learning, the Society should develop such resources.

**Supplementary Information:**

The online version contains supplementary material available at 10.1186/s12909-026-08814-7.

## Background

The United Kingdom Medical Licensing Assessment (UKMLA) is a new national examination that all UK medical students must pass before graduation. It assesses the knowledge and skills set out in the Outcomes for Graduates, ensuring a consistent standard for safe medical practice [1, 2]. The UKMLA comprises two components: the Applied Knowledge Test (AKT), an on-screen multiple-choice exam, and the Objective Structured Clinical Examination (OSCE), which evaluates clinical and professional skills. In 2021, the General Medical Council published a revised content map outlining the material assessed in both components.

Medical students frequently supplement university teaching with external digital resources [3]. With the introduction of the UKMLA, there is a growing opportunity to develop resources that cater to students nationwide. Among paid tools, question banks are the most widely used [4, 5], helping students address gaps in teaching, practice exam-style questions, and receive immediate feedback. Free platforms such as YouTube© are also widely used [6–8]. Students commonly use them to revisit previously taught material [6] or to explore topics such as radiology and pathology in greater depth within the undergraduate curriculum [7, 8]. Despite the variable quality of available content, some evidence suggests these resources can improve test performance [9, 10]. Spaced-repetition tools like Anki are widely adopted and support long-term knowledge retention [11, 12]. More recently, medical students have begun to use artificial intelligence tools [13]. Popular large language models, such as ChatGPT©, are being used to simulate patient interactions [14], and deep learning systems have been developed to automatically assess medical trainees [15].

Recognising the growing reliance on digital learning, the Royal Society of Medicine identified an opportunity to support UKMLA preparation through accessible online materials. We therefore piloted a 24-part online lecture series to provide clinical-year medical students with a concise revision resource which could be used alongside their existing medical school organised teaching to prepare for the UKMLA. This study aims to determine which types of resources the Society should prioritise in future development.

## Methods

The authors designed a 24-lecture series covering high-yield conditions from the UKMLA curriculum map. A post-CCT, GMC-registered specialist selected the high-yield topics and defined the relevant learning objectives for each lecture. Each lecture was planned to last one hour, including questions and answers, and two lectures took place each week between April 2024 and June 2024. Each individual lecture and its corresponding learning objectives can be found in supplementary Table 1. The lecture series was marketed and delivered by the Royal Society of Medicine’s Academic Section. Marketing primarily involved distributing information through social media and group chats across the UK. Our target audience was UK medical students in their clinical years of study. Medical students were required to pay £5 for access to the whole series and the recordings. Students were able to pay for lectures by signing up through Eventbrite©. Each of the 24 lectures was delivered using the Google© Meets online platform. The lectures were also recorded using the same software.

An online survey was created using Google© Forms, here after referred to as The Student Educational Resource Preferences Survey (SERPS). The form was advertised throughout the sessions and explored the resources students used to prepare for the UKMLA (supplementary Table 2).

Lower numerical responses indicated negative feedback and higher responses indicated positive feedback (0 = least satisfied, 10 = most satisfied). Students provided their name and email address solely to prevent duplicate submissions.

For each question, data were analysed using descriptive statistical methods, with the mean and mode calculated to characterise the distribution of responses.

## Results

A total of 200 tickets were sold. Attendance numbers for each individual lectures can be found in supplementary Table 3.

SERPS was answered by 71 participants (Table 1). Most participants were UK medical students and from 23 different universities answered the questionnaire. There were 8 participants who were studying at an overseas medical school.

**Table 1:** Demographics of participants who answered the SERPS

Row Labels	Intercalating	Year 1	Year 2	Year 3	Year 4	Year 5	Year 6	Grand Total
Anglia Ruskin University School of Medicine				3				3
Aston University Medical School				2	2			4
Brighton and Sussex Medical School		2		1	1			4
Cardiff University School of Medicine				1				1
Hull York Medical School					2			2
Imperial College London Faculty of Medicine		1						1
King’s College London GKT School of Medical Education				4	1		1	6
Lancaster University Medical School				1				1
Newcastle University School of Medical Education				2				2
Plymouth University Peninsula Schools of Medicine and Dentistry				2				2
Queen Mary University of London				1				1
Queen’s University Belfast School of Medicine				4		1		5
St George’s, University of London		1		1	1			3
Ulster University, School of Medicine				1	1			2
University College London					4			4
University of Bristol Medical School				1				1
University of Buckingham Medical School			1	3	3			7
University of Cambridge School of Clinical Medicine						1		1
University of Leeds School of Medicine					1			1
University of Leicester Medical School					4			4
University of Liverpool School of Medicine	1		1	2				4
University of Manchester Medical School				1				1
University of Nottingham School of Medicine				1	2			3
Other		1	5		1	1		8
Grand Total	1	5	7	31	23	3	1	71

**Fig. 1:**
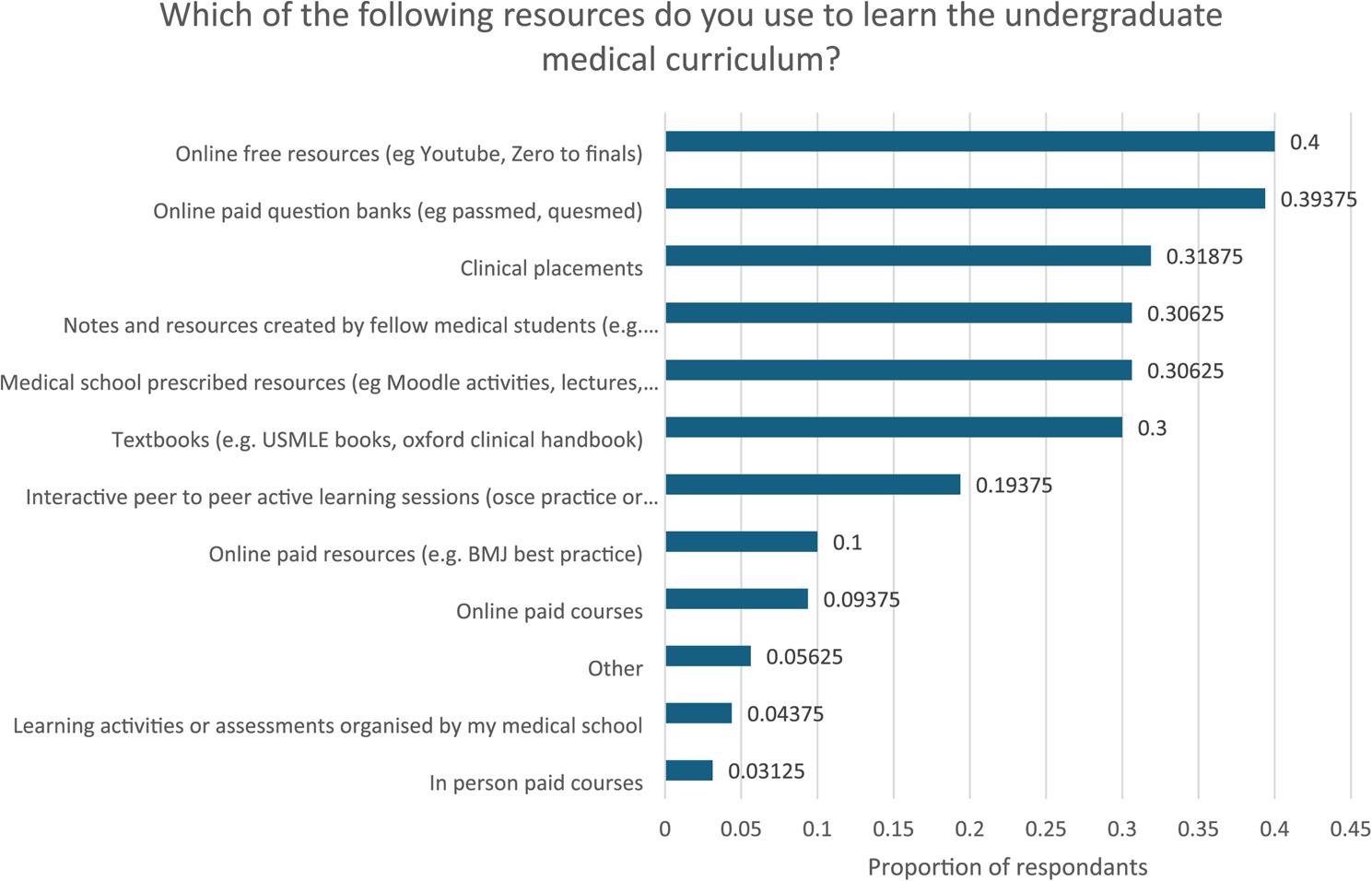
Proportion of respondents responding to individual options to the SERPS question “How would you prefer the content covered in this lecture to be delivered question?”. *N*=71

The most used resource mentioned by medical students (Fig. 1) was free online resources, with 64 participants stating they used these for learning the undergraduate medical curriculum. The second most used resource was paid online question banks, with 63 students reporting using them. The third most used resource was clinical placements, with 51 students reporting using them to learn the undergraduate medical curriculum.

**Fig. 2:**
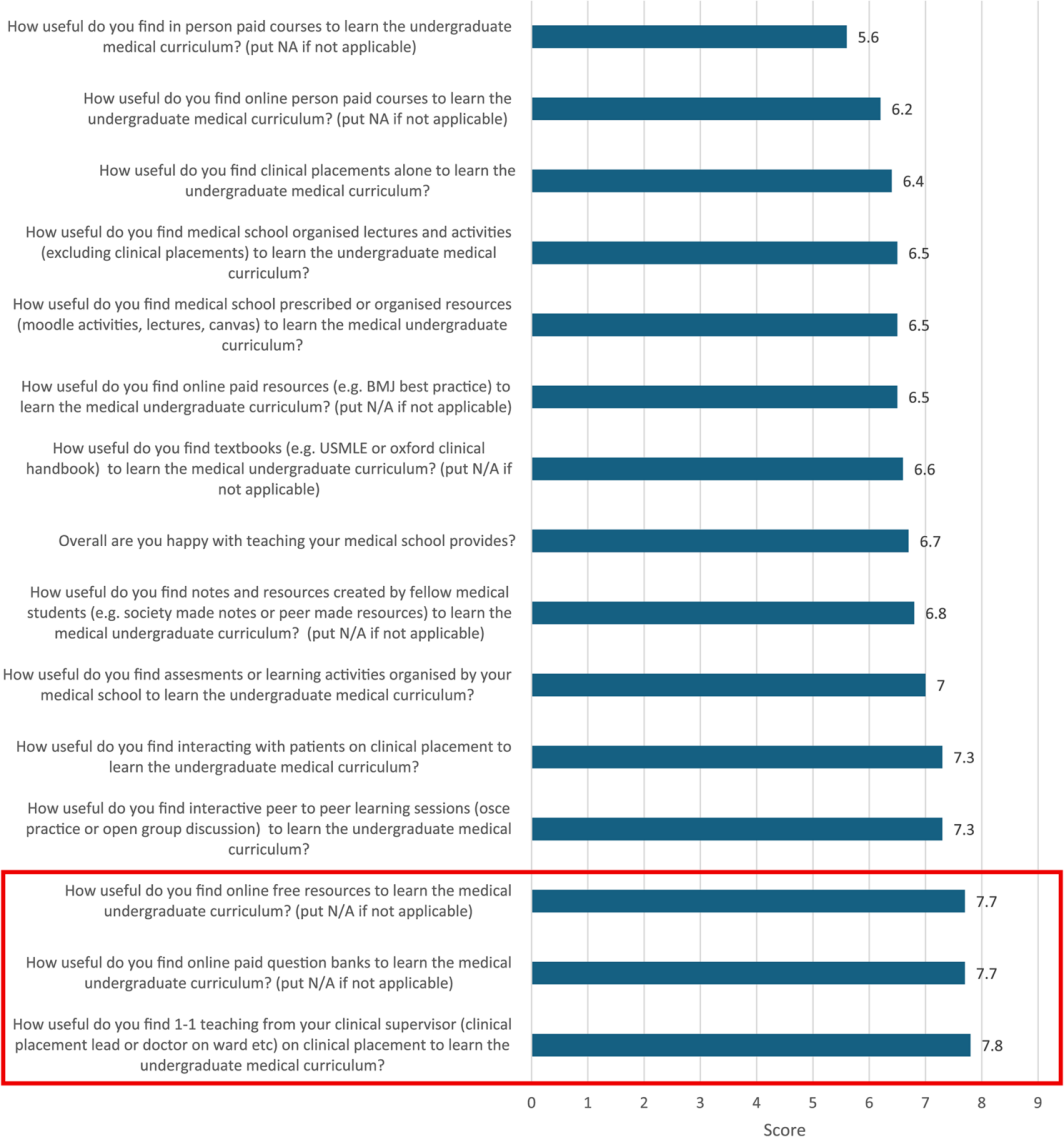
Mean response scores for Likert scale items in the SERPS arranged in order of lowest to highest score, red rectangle shows the items scoring top three. *N*=71

When rating the usefulness of different learning methods, the most useful was one-to-one teaching from clinical supervisors, which participants rated an average of 7.8/10 (Fig. 2). This was closely followed by paid online question banks and free online resources; both rated an average of 7.7/10 in usefulness. The next most useful resources were interacting with patients on placement and interactive peer-to-peer learning, both scoring 7.3/10 on average.

**Fig. 3:**
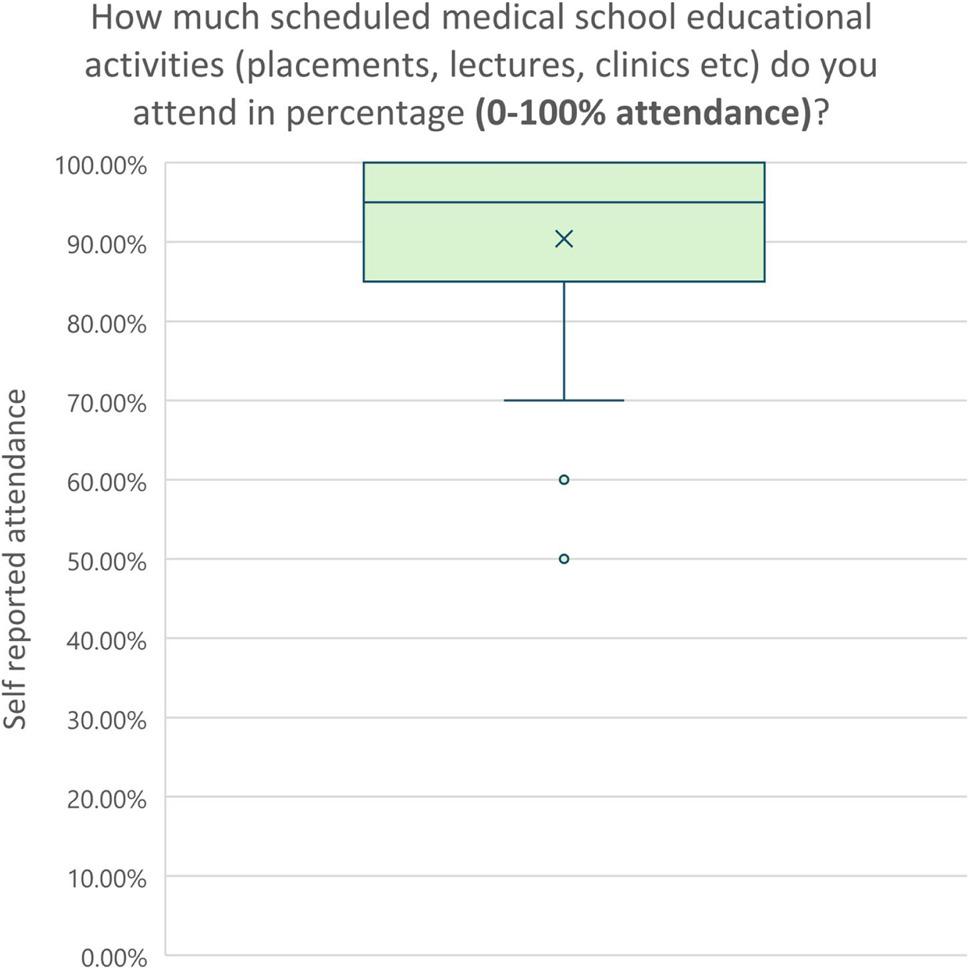
Percentage distribution to the SERPS question “How much scheduled medical school educational activities (placements, lectures, clinics etc) do you attend in percentage (0-100% attendance)?”. *N*=71

Medical students also reported a high percentage of attendance on clinical placements (Fig. 3), with a median of 95% (question 19).

**Fig. 4:**
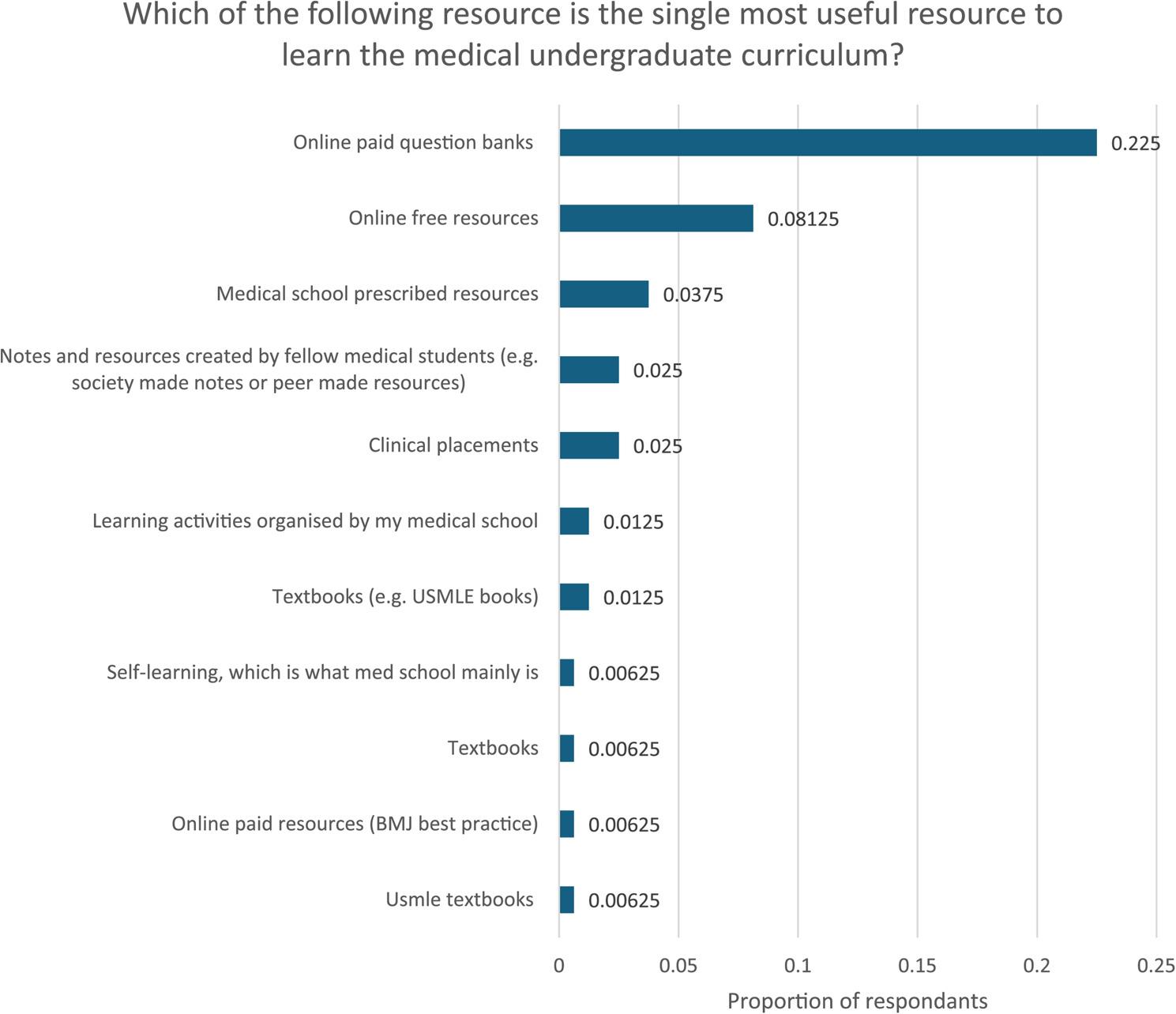
Proportion of respondents responding to individual option to the SERPS question “Which of the following resource is the single most useful resource to learn the medical undergraduate curriculum?”. *N* =71

These results were reflected when medical students were asked which was the single most useful resource for their learning (Fig. 4). Paid online question banks were reported as the single most useful (36/71), followed by free online resources (13/71).

## Discussion

This study represents the first initiative by the Royal Society of Medicine’s Academic Section to deliver a national, lecture-based teaching series aimed at medical students across the United Kingdom. Our objective was to develop a concise revision resource that would be useful for clinical medical students preparing for their UKMLA. By ensuring sessions were delivered by a post-CCT doctor, our lectures were of high quality and contained accurate information, often lacking in online resources.

Our study aimed to identify which resources medical students use to prepare for the UKMLA, to inform the society’s future priorities. In terms of revision resources, our questionnaire confirmed that students primarily rely on commercial question banks (e.g., Passmed©, Quesmed©) and free online platforms (e.g., Zero to Finals©). The popularity of question banks is consistent with international literature [16–18], with peer recommendations and the provision of exam-style practice questions cited as key drivers of use [18]. Understanding why medical students use question banks was not answered by our study but it has been suggested peer recommendations of the resource plays a significant role in medical students’ utilising the resource as well as the simulated exam practice question banks provide [18]. The use of question banks being helpful is also supported by studies which demonstrate question bank usage is correlated with better academic performance [19]. Although question banks and online resources were most preferred, the third most preferred method of learning is clinical placements or medical school resources, hence medical school prescribed activities do provide limited value. Medical students find interacting with patients on clinical placement and receiving 1–1 teaching from clinical supervisor useful. The one-to-one teaching medical students receive during clinical placements plays a vital role in delivering effective clinical supervision, as highlighted by many studies [19–21]. Despite the importance of supervision, it may not be ranked as highly as other learning resources, as clinical supervisors often have competing service commitments that dilute their educational responsibilities, making consistent supervision difficult to achieve [22–32]. Medical students nonetheless reported a median attendance of 95% for scheduled activities, suggesting they generally take prescribed teaching seriously. That said, attendance is compulsory for all medical school organised placements hence medical students may have reported a high attendance. Multiple studies have noted declining attendance at lectures [25–27]. Students often prefer recorded online lectures over in-person sessions, as they provide greater efficiency and flexibility [28–30]. Since the COVID-19 pandemic, many medical schools have shifted towards online formats [31], which may partly explain the high reported attendance rates.

Students were least satisfied with institution-prescribed resources, which are typically textbook- or lecture-based. This suggests a widening gap between traditional formats and modern learners’ preferences for digital, interactive methods. Similarly, in-person revision courses were poorly rated, likely due to limited accessibility and geographic constraints.

Our series successfully reached a national cohort of clinical-year students, spanning 23 UK medical schools. Although only a small number of students from each institution responded, the findings suggest that medical students are receptive to an initiative of this scope. This highlights the value of continuing to explore their interest in similar national programmes.

A key limitation of this study is the potential for inherent selection bias. Participation in the questionnaire was voluntary, and the lecture series itself was marketed and delivered in an online format. These likely attracted students who were predisposed to favour online learning, thereby skewing responses towards more positive evaluations of the lecture series and a preference for digital resources. Consequently, our results should be interpreted as most applicable to a cohort of motivated, digitally engaged learners rather than the entire medical student population.

The SERPS survey featured several broadly framed questions that are open to interpretation. Specifically, questions referencing the medical undergraduate curriculum are highly subjective, as what constitutes the curriculum for one student may differ significantly for another. Consequently, the resulting scores are likely influenced by this variability in individual interpretation.

It is important to acknowledge that our surveys were completed by 71 respondents, representing a modest sample size. This introduces the potential for responder bias and means our findings should not be generalised to the entire UK medical student population. The modest sample size decreases the power of this study, making it challenging to perform statistical tests and understand if differences in values are truly significant. Therefore, it is essential to evaluate the content of the lectures and re-conduct the SERPS survey when a more representative sample is available.

Nevertheless, our work offers several contributions. To our knowledge, this is the first study to explore UK medical students’ preferences for learning resources specifically in the context of preparing for the UKMLA, a new national examination with limited published data on how students are approaching revision. Finally, by asking students to directly compare university teaching, clinical placements, question banks, and online resources, our study provides relative benchmarking across resource types rather than isolated ratings. We therefore view this study as an exploratory step that highlights early national trends and generates hypotheses for future large-scale, representative studies.

Another limitation was the absence of data on artificial intelligence (AI) tools, which have rapidly gained prominence since the survey was designed in 2022 and data were collected in early 2023. Future studies should explore how emerging AI technologies can further support medical students preparing for the UKMLA.

Despite the limitations of our study, it is apparent that the Society should continue to deliver online content. To further help clinical medical students, we should focus on developing other digital resources. One study that could inspire future initiatives is that undertaken by Schwartzman [32]. The study partnered students and university faculty members to create high-quality, reliable question banks. Adopting a similar model could allow us to generate resources that align closely with students’ preferred methods of learning. Further studies are planned to address what is the best online delivery format.

## Conclusion

Our study demonstrates that a centrally delivered, national teaching series can be well received and valued by UK medical students. However, to optimise its impact, future initiatives could complement lectures with additional digital resources, particularly high-quality question banks developed in collaboration with faculty and students. Ultimately, combining the rigour of institution-led resources with the accessibility and adaptability of modern digital tools may offer the most effective approach to supporting students preparing for the UKMLA.

## Supplementary Information

Supplementary Material 1.

Supplementary Material 2.

Supplementary Material 3.

## Data Availability

The datasets used and/or analysed during the current study are available from the corresponding author on reasonable request.

## Abbreviations

UKMLA: United Kingdom Medical Licencing Assessment
CCT: Certificate of Completion of Training
