# Supplementary material for: Understanding the ideal resources to study the UKMLA
Source: BMC Med Educ. 2026 Feb 23;26:517. doi: 10.1186/s12909-026-08814-7 (PMC13036964; doi:10.1186/s12909-026-08814-7)
Supplement: Supplementary file 1 — Supplementary Material 1. [file 12909_2026_8814_MOESM1_ESM.docx]

| **Lecture number** | **Lecture title** | **Lecture learning objectives** |
| --- | --- | --- |
| 1 | Cardiology 1 | Tachycardias and ECGs - An approach to analysing ECGs. - Tachycardias - AF, atrial flutter, SVT vs VTs, VT, VF, torsades de pointes (identification on ECG and Mx) - AF: aetiology, risk factors, Sx, Ix, Mx (rate vs rhythm control), stroke risk and CHADsVASc - Mx of tachycardias in an unstable patient (i.e., DC cardioversion) - ABCDE approach |
| 2 | Cardiology 2 | Heart Failure  - aetiology, risk factors, NYHA classification - Acute vs chronic - HFrEF vs HFpEF - Systolic vs diastolic - High output vs low output - Left vs right - Symptoms, investigations, complications, management |
| 3 | Renal medicine | AKI 1. Criteria for AKI (NICE) 2. Risk factors 3. Causes (pre-renal, renal, post-renal) 4. Presentation (signs and symptoms) 5. Investigations (urinalysis, ultrasound) 6. Management (3 step management, drugs they are on, hyperkalemia, fluid support) 7. Staging AKIs  CKD 1. Causes (risk factors) 2. Presentation/features 3. Investigations (eGFR, ACR, urine dipstick, ultrasound) 4. Staging of CKD (G and A score) 5. Criteria for referral to specialist 6. Management (slowing progression, treating complications (met acidosis, anaemia, bone disease, renal failure) treating hypertension) 7. Anaemia of CKD (causes, management) 8. Bone disease of CKD (causes, management) |
| 4 | Rheumatology | OA vs RA vs sero-negative arthritis - aetiology, risk factors, causes - Sx: key focus on differentiating between the different conditions  - Ix and Dx: key differences in X-Rays and joints involved; antibodies - Mx - Prognosis |
| 5 | Sexual Health | HIV, chlamydia, syphillis, gonorrhoea (- aetiology, risk factors, causes, - Sx: key focus on differentiating between these STIs (rashes? discharge? color of discharge?, infection disease and managmenet, prognosis) |
| 6 | Infectious Medicine | Malaria, TB, Lyme Disease - a history taking approach to the "sick traveller" - aetiology, risk factors, causes - symptoms: key focus on differentiating between these  - investigations and differentials  - management  - Prognosis  - legal aspects e.g. notifiable disease |
| 7 | Respiratory: Asthma and COPD | COPD  1. Symptoms + presentation (+ risk factors) + pathophysiology  2. Diagnosis + risk factors (spirometry, CXR, CT, FBC)  3. Management (general advice, NICE steps, LTOT criteria)  4. Exacerbations (features, when to admit to hospital, causes, management)  ASTHMA 5. Pathophysiology  6. Symptoms, precipitants, risk factors, signs  7. Diagnosis + investigations  8. Management steps, stepping down treatment 9. Types of attacks (criteria for each type)  10. Management of attacks, when to admit, discharge criteria |
| 8 | Respiratory: Respiratory Infections | 1. Pneumonia (CURB65, types, symptoms and signs, investigations, management, discharge criteria, complications of pneumonia, atypical pneumonia) 2. URTI 3. LRTI |
| 9 | Dermatology | Acne, atopic dermatitis (eczema), keratosis pilaris, psoriasis, rosacea, urticaria, skin infections (impetigo, cellulitis, abscesses, necrotising fasciitisi), viral skin infections, blistering skin disorders |
| 10 | Respiratory: PE and and pneumothorax | Pneumothorax  1. Features & risk factors  2. Causes (spontaneous, lung disease, CT disease, iatrogenic, trauma)  3. Investigations  4. Management (primary, secondary, iatrogenic)  5. Discharge advice  6. Tension pneumothorax (signs, management: cannula, chest drain)   PE  1. Features  2. Investigations (NICE workflow, ECG) 3. Management with anticoagulants + special cases |
| 11 | Gastroenterology: Upper GI disorders | Upper GI dx: hiatial hernia, GORD, ulcers, gastrointestinal perforation - risk factors, epidemiology  - pathogenesis, signs and symptoms, investigations - management, complications |
| 12 | Gastroenterology: Lower GI disorders | Lower GI dx: Crohn's + Ulcerative colitis (IBDx), IBS, malabsorption dx, hemorrhoids, fissues - risk factors, epidemiology  - pathogenesis, signs and symptoms, investigations - management, complications - diffrenciating between crohn's, UC, and diffrenciating between IBD and IBS - histological features and differences between different IBDx on imaging |
| 13 | Gastroenterology: Hepatobiliary disorders | Hepatobiliary dx: pancreatitis, cholecystitis, cholangitis, gallstones + biliary colic, hepatitis, liver failure, cirrhosis - risk factors, epidemiology  - pathogenesis, signs and symptoms, investigations - management, complications |
| 14 | X-rays | MSK X-Rays - approach to interpretation, importance of multiple views, fracture classification (transverse, linear, spiral, oblique, etc.), identification and description of upper and lower limb fractures, signs of arthritis, soft tissue injury + any other relevant pathology |
| 15 | Neurology: Neurovascular disorders | Neurovascular dx: TIAs; strokes (ischameic and hemorrhagic, hameorrhages (including identification on CTs)- risk factors, epidemiology. - pathogenesis, signs and symptoms, investigations. - management, complications. - localizing stroke location based on focal neurological signs. Key conditions: Head injury, trauma, strokes, transient ischemic attacks, blackouts and faints, headache? |
| 16 | Neurology: CNS disorders | CNS dx: infectious dx; cranial nerve palsies; epilepsy - risk factors, epidemiology - pathogenesis, signs and symptoms, investigations - management, complications - focal neurological signs in individual CN palsies Key conditions: meningitis, encephalitis, brain abcesses, bell's palsy |
| 17 | Neurology: PNS disorders | PNS dx: neuropathies, radiculopathy, spinal cord compression and injury, weakness - risk factors, epidemiology  - pathogenesis, signs and symptoms, investigations - management, complications - focal signs of specific spinal nerve compressions, and differenciating between spinal cord compression and spinal stenosis |
| 18 | Neurology (MNDx) | Motor neuron dx: GBS, muscular dystrophies (including ALS) - risk factors, epidemiology  - pathogenesis, signs and symptoms, investigations - management, complications |
| 19 | Ophthalmology | Glaucoma, visual field defects, optic neuritis, macular degeneration - linking visual field defects to specific stroke lesions  - acute vs chronic glaucoma: aetiology, risk factors, Sx, Ix, Dx, Mx, prognosis  - retinal detachment: Sx and emphasizing that it is a medical emergency! - optic neuritis  - Differentials of: sudden vision loss; gradual vision loss; diplopia; red eye; painful eye |
| 20 | Endocrinology 1 | 1. Thyroid function tests and diagnoses + thyroid scintigraphy  2. Hypothyroidism (Causes, Features/presentation, Management) 3. Thyrotoxicosis (Features/presentation, Causes, Management) 4. Thyroid storm (Precipitating events, Clinical features, Management) 5. Physiology of adrenals (zones and secretions) 6. Cushings (Presentation, diagnosis, management)  7. Addisons (Presentation, diagnosis, management) 8. Conn’s (Presentation, diagnosis, management) 9. Phaeochromocytoma (Presentation, diagnosis, management) |
| 21 | Endocrinology 2 | 1. Pathophysiology of T1DM and T2DM  2. Brief mention of the other types of diabetes  3. Diagnostic criteria and monitoring tests (T1vsT2) with normal and abnormal values  4. short term and long term complications (T1vsT2)  5. Diabetic emergencies (diagnosis + presentation, management + resolution) 1. Hypoglycemia  2. DKA  3. HHS 6. Diabetic drugs (NICE guidelines for indications, side effects, contraindications, mechanism of action) (Metformin, SGLT2 inhibitors, DPP4 inhibitors, Sulfonylureas, TZDs, GLP1, Insulin |
| 22 | ENT | Conditions: Otitises media and externa, OSA, BPPV, Meniere's dx, infectious mononucleosis, tonsillitis, epiglottitis, croup. - risk factors, epidemiology. - pathogenesis, signs and symptoms, investigations. - management, complications |
| 23 | Psychiatry 1 | Depression, anxiety, schizophrenia, bipolar disorder - aetiology, risk factors, Sx, Ix, Dx and Mx for all  Schizophrenia specific: - psychosis vs schizophrenia - first rank; positive vs negative Sx - schizophrenia vs schizoid PD vs schizotypical PD - Mental Health Act sections + mental capacity |
| 24 | Psychiatry 2 | Psychiatric Drugs - typical vs atypical antipsychotics - SSRIs, SNRIs, NaSSA, TCA, NDRI - benzodiazepines - lithium/mood stabilisers - neuroleptic malignant syndrome; serotonin syndrome - For each, MOA, indications, side effects |
| 25 | Sexual Health | HIV, chlamydia, syphilis, gonorrhoea (- aetiology, risk factors, causes, - Sx: key focus on differentiating between these STIs (rashes? discharge? color of discharge?, infection disease and managmenet, prognosis) |

Supplementary table 1: States the order of the lectures delivered, respective lecture titles and learning objectives of individual sessions.
