# Supplementary material for: Understanding the ideal resources to study the UKMLA
Source: BMC Med Educ. 2026 Feb 23;26:517. doi: 10.1186/s12909-026-08814-7 (PMC13036964; doi:10.1186/s12909-026-08814-7)
Supplement: Supplementary file 2 — Supplementary Material 2. [file 12909_2026_8814_MOESM2_ESM.docx]

| **Question number**  Supplementary table 2: The question number, variables, question types and options available on the SERPS. | **Variable** | **Question type** | **Options available** |
| --- | --- | --- | --- |
| 1 | Medical school and location of medical school | Single choice | University of Aberdeen School of Medicine and Dentistry  Anglia Ruskin University School of Medicine  Aston University Medical School  Queen Mary University of London  University of Birmingham College of Medical and Dental Sciences  Brighton and Sussex Medical School  University of Bristol Medical School  University of Buckingham Medical School  University of Cambridge School of Clinical Medicine  Cardiff University School of Medicine  University of Dundee School of Medicine  Edge Hill University Medical School  The University of Edinburgh Medical School  University of Exeter Medical School  University of Glasgow School of Medicine  Hull York Medical School  Imperial College London Faculty of Medicine  Keele University School of Medicine  Kent and Medway Medical School  King's College London GKT School of Medical Education  Lancaster University Medical School  University of Leeds School of Medicine  University of Leicester Medical School  University of Liverpool School of Medicine  London School of Hygiene & Tropical Medicine  University of Manchester Medical School  Newcastle University School of Medical Education  University of East Anglia, Norwich Medical School  University of Nottingham School of Medicine  University of Nottingham - Lincoln Medical School  University of Oxford Medical Sciences Division  Plymouth University Peninsula Schools of Medicine and Dentistry  Queen's University Belfast School of Medicine  University of Sheffield Medical School  University of Southampton School of Medicine  University of St Andrews School of Medicine  St George's, University of London  University of Sunderland School of Medicine  Swansea University Medical School  University of Central Lancashire School of Medicine  University of Warwick Medical School  Brunel University London, Brunel Medical School  Ulster University, School of Medicine  University of Chester Medical School  Three Counties Medical School  University College London  North Wales Medical School, Bangor University |
| 2 | Year | Single choice | Year 1  Year 2  Year 3  Year 4  Year 5  Year 6  Intercalation year |
| 3 | Which of the following resources do you use to learn the undergraduate medical curriculum | Multiple choice | Online paid question banks (e.g. Passmed^©^, Quesmed^©^) |
|  |  |  | Online free resources (e.g. YouTube^©^, Zero to Finals^©^) |
|  |  |  | Textbooks (e.g. USMLE books, oxford clinical handbook) |
|  |  |  | Medical school prescribed resources (e.g. Moodle activities, lectures, canvas,) |
|  |  |  | In person paid courses |
|  |  |  | Online paid courses |
|  |  |  | Notes and resources created by fellow medical students (e.g. society made notes or peer made resources) |
|  |  |  | Clinical placements |
|  |  |  | Online paid resources (e.g. BMJ best practice) |
|  |  |  | Learning activities or assessments organised by my medical school |
|  |  |  | Learning activities or assessments organised by my medical school.  Interactive peer to peer active learning sessions (OSCE practice or open group discussion) |
|  |  |  |  |
| 4 | If your resources that you used are not listed above – write them down here | Free text |  |
| 5 | How useful do you find online paid question banks to learn the medical undergraduate curriculum? **(put N/A if not applicable)** | Likert-scale | 1 (not useful) – 10 (very useful), N/A |
| 6 | How useful do you find **online free resources** to learn the medical undergraduate curriculum? **(put N/A if not applicable)** | Likert-scale | 1 (not useful) – 10 (very useful), N/A |
| 7 | How useful do you find **online paid resources**(e.g. BMJ best practice) to learn the medical undergraduate curriculum? **(put N/A if not applicable)** | Liker-scale | 1 (not useful) – 10 (very useful), N/A |
| 8 | How useful do you find **textbooks**(e.g. USMLE or oxford clinical handbook) to learn the medical undergraduate curriculum? **(put N/A if not applicable)** |  | 1 (not useful) – 10 (very useful), N/A |
| 9 | How useful do you find **medical school prescribed or organised resources** (Moodle activities, lectures, canvas) to learn the medical undergraduate curriculum? |  | 1 (not useful) – 10 (very useful) |
| 10 | How useful do you find **notes and resources created by fellow medical students** (e.g. society made notes or peer made resources) to learn the medical undergraduate curriculum? **(put N/A if not applicable)** |  | 1 (not useful) – 10 (very useful), N/A |
| 11 | How useful do you find**clinical placements alone** to learn the undergraduate medical curriculum? |  | 1 (not useful) – 10 (very useful) |
| 12 | How useful do you find **medical school organised lectures and activities**(excluding clinical placements) to learn the undergraduate medical curriculum? |  | 1 (not useful) – 10 (very useful) |
| 13 | How useful do you find **in person paid courses** to learn the undergraduate medical curriculum? **(put NA if not applicable)** |  | 1 (not useful) – 10 (very useful), N/A |
| 14 | How useful do you find online person paid courses to learn the undergraduate medical curriculum? **(put NA if not applicable)** |  | 1 (not useful) – 10 (very useful), N/A |
| 15 | How useful do you find **interactive peer to peer learning sessions**(OSCE practice or open group discussion) to learn the undergraduate medical curriculum? |  | 1 (not useful) – 10 (very useful), N/A |
| 16 | How useful do you find **1-1 teaching from your clinical supervisor** (clinical placement lead or doctor on ward etc) on **clinical placement**to learn the undergraduate medical curriculum? |  | 1 (not useful) – 10 (very useful) |
| 17 | How useful do you find **interacting with patients** on clinical placement to learn the undergraduate medical curriculum? |  | 1 (not useful) – 10 (very useful) |
| 18 | How useful do you find **assessments or learning activities** organised by your **medical school**to learn the undergraduate medical curriculum? |  | 1 (not useful) – 10 (very useful) |
| 19 | How much scheduled medical school educational activities (placements, lectures, clinics etc) do you attend in percentage **(0-100% attendance)**? | Free text |  |
| 20 | Which of the following resource is the single most useful resource to learn the medical undergraduate curriculum? |  | Online paid question banks (e.g. Passmed^©^, Quesmed^©^)  Online free resources (eg YouTube^©^, Zero to Finals^©^)  Textbooks (e.g. USMLE books)  Medical school prescribed resources (e.g. Moodle activities, lectures, canvas)  In person paid courses  Online paid courses  Notes and resources created by fellow medical students (e.g. society made notes or peer made resources)  Clinical placements  Learning activities or assessments organised by my medical school.  Online paid resources (BMJ best practice)  Peer to peer learning sessions (OSCE practice or open group discussion)  Other |
| 21 | Overall are you happy with teaching your medical school provides? |  | 1 (not useful) – 10 (very useful) |
| 22 | **Please provide any comments not covered above, or suggestions you would like to make on this topic** | Free text |  |
