# Supplementary material for: Understanding the ideal resources to study the UKMLA
Source: BMC Med Educ. 2026 Feb 23;26:517. doi: 10.1186/s12909-026-08814-7 (PMC13036964; doi:10.1186/s12909-026-08814-7)
Supplement: Supplementary file 3 — Supplementary Material 3. [file 12909_2026_8814_MOESM3_ESM.docx]

| **Lecture number** | **Lecture title** | **Attendee number** |
| --- | --- | --- |
| 1 | Cardiology 1 | 37 |
| 2 | Cardiology 2 | 30 |
| 3 | Renal medicine | 28 |
| 4 | Rheumatology | 26 |
| 5 | Sexual Health | 1 |
| 6 | Infectious Medicine | 23 |
| 7 | Respiratory: Asthma and COPD | 24 |
| 8 | Respiratory: Respiratory Infections | 27 |
| 9 | Dermatology | 29 |
| 10 | Respiratory: PE and and pneumothorax | 24 |
| 11 | Gastroenterology: Upper GI disorders | 23 |
| 12 | Gastroenterology: Lower GI disorders | 25 |
| 13 | Gastroenterology: Hepatobiliary disorders | 22 |
| 14 | X-rays | 30 |
| 15 | Neurology: Neurovascular disorders | 33 |
| 16 | Neurology: CNS disorders | 33 |
| 17 | Neurology: PNS disorders | 34 |
| 18 | Neurology (MNDx) | 33 |
| 19 | Ophthalmology | 12 |
| 20 | Endocrinology 1 | 28 |
| 21 | Endocrinology 2 | 29 |
| 22 | ENT | 29 |
| 23 | Psychiatry 1 | 33 |
| 24 | Psychiatry 2 | 34 |
| 25 | Sexual Health | 1 |

Supplementary table 3: States the number of attendees for each individual lecture.
